# Three-dimensional echocardiographic assessment of Chiari’s network relationship with the left ventricular false tendon

**DOI:** 10.1186/s43044-022-00287-5

**Published:** 2022-06-15

**Authors:** Mutlu Cagan Sumerkan, Sukru Cetin, Fusun Behramoglu Helvaci, Sendag Satilmis Yaslikaya, Umut Karabay, Turgun Hamit, Ahmet Gurdal, Mehmet Agirbasli, Omer Alyan

**Affiliations:** 1 Department of Cardiology, University of Health Sciences Turkey, Sisli Hamidiye Etfal Training and Research Hospital, Istanbul, Turkey; 2 Department of Internal Medicine, University of Health Sciences Turkey, Sisli Hamidiye Etfal Training and Research Hospital, Istanbul, Turkey; 3 Department of Cardiology, Faculty of Medicine, Istanbul Medeniyet University, Istanbul, Turkey

**Keywords:** Abnormalities, Cardiovascular, Chiari’s network, Three-Dimensional echocardiography, False tendon

## Abstract

**Background:**

Left ventricular false tendon (LVFT) is a fibromuscular band crossing the left ventricular cavity. And Chiari’s network (CN) is a highly mobile, mesh-like, echogenic structure in right atrium. In this study, we aimed to evaluate the coexistence of LVFT in patients with CN. CN patients were examined with live/real-time three-dimensional transthoracic echocardiography (TTE) for visualization of LVFT.

**Results:**

This is a single-center prospective study of 49 patients with CN. In literature studies, the average ratios of LVFT were 22% in the normal population. In our study, an increased ratio of LVFT (*n* = 31, 63.3%) was found in CN patients evaluated with a three-dimensional TTE (63.3% versus 22%) (*p* = 0.01). The interatrial septal aneurysm was found in 31 (63.3%) patients with CN. And, the positive contrast echocardiography examination was determined in 22 (61.1%) patients with CN.

**Conclusions:**

Our study reveals that CN is associated with LVFT and is also associated with cardiac anomalies like an interatrial septal aneurysm, and atrial septal defect. And LVFT can be evaluated better with three-dimensional TTE than with traditional two-dimensional TTE. Patients with CN should be evaluated more carefully by three-dimensional echocardiography as they can be in synergy in terms of the cardiac pathologies they accompany.

**Supplementary Information:**

The online version contains supplementary material available at 10.1186/s43044-022-00287-5.

## Background

**Fig. 1:**
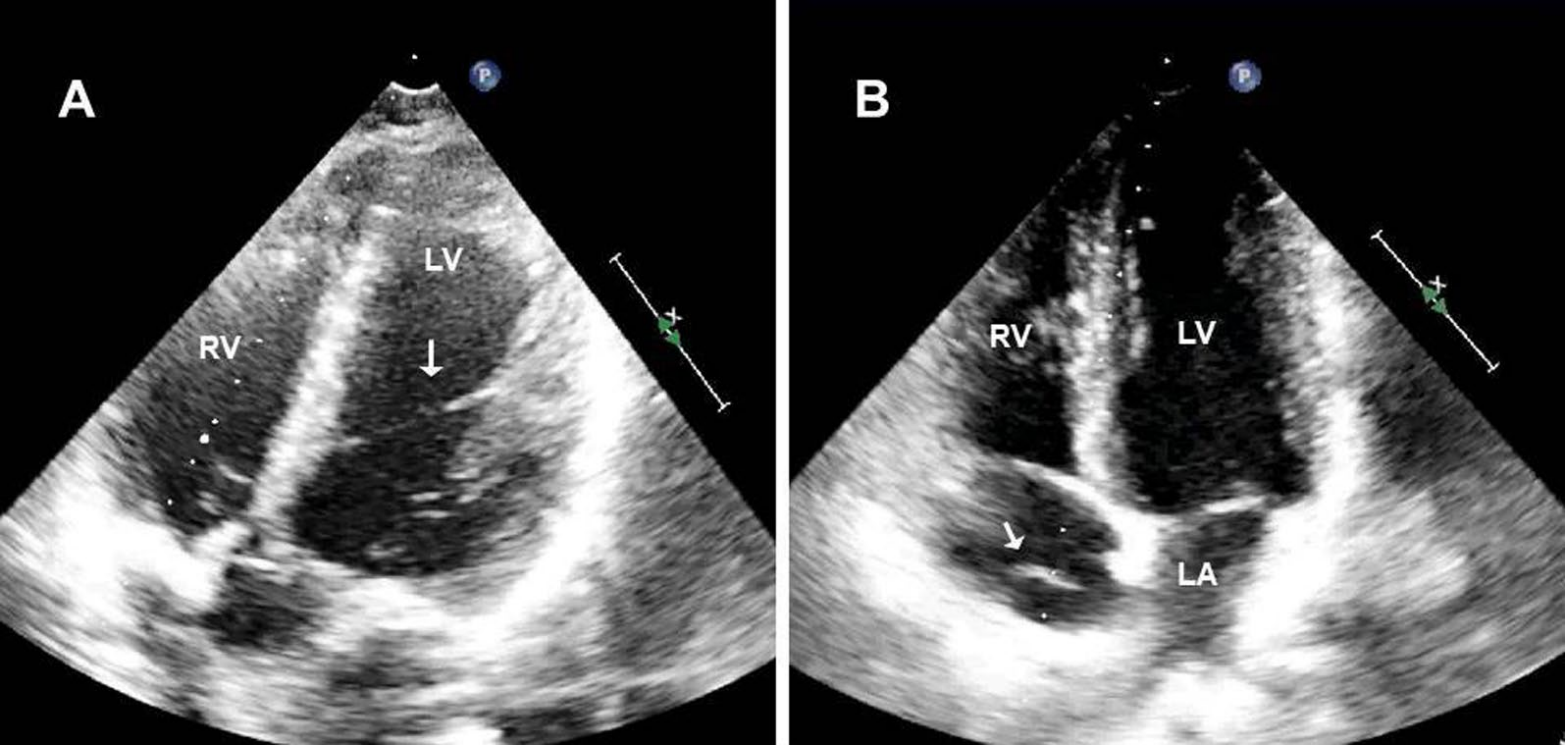
**A** Modified apical five-chamber imaging shows the left ventricular false tendon extending between the interventricular septum and the left ventricle lateral wall. **B** In the four-chamber imaging of the same patient, the Chiari’s network is observed in the right atrium

Chiari’s network (CN) is seen as a mobile, reticulated structure in many localizations of the right atrium (Fig. 1, Additional file 1: Video 1). It is the right leaflet remnant of the sinus venous and directs blood flow from the inferior vena cava to the interatrial septum in embryological life [1]. It was first defined by Hans Chiari [1]. Generally, it has no clinical findings. However, autopsy [1] and echocardiography [2] studies have shown that the incidence of clinically important cardiac abnormalities such as patent foramen ovale (PFO), atrial septal defect (ASD), and interatrial septal aneurysm (IASA) is higher in patients with CN [3]. As with other right-sided heart structures, CN could be a potential site for the development of infective endocarditis [4]. Catheter entrapment may also happen around the CN during intracardiac procedures [5].

**Fig. 2:**
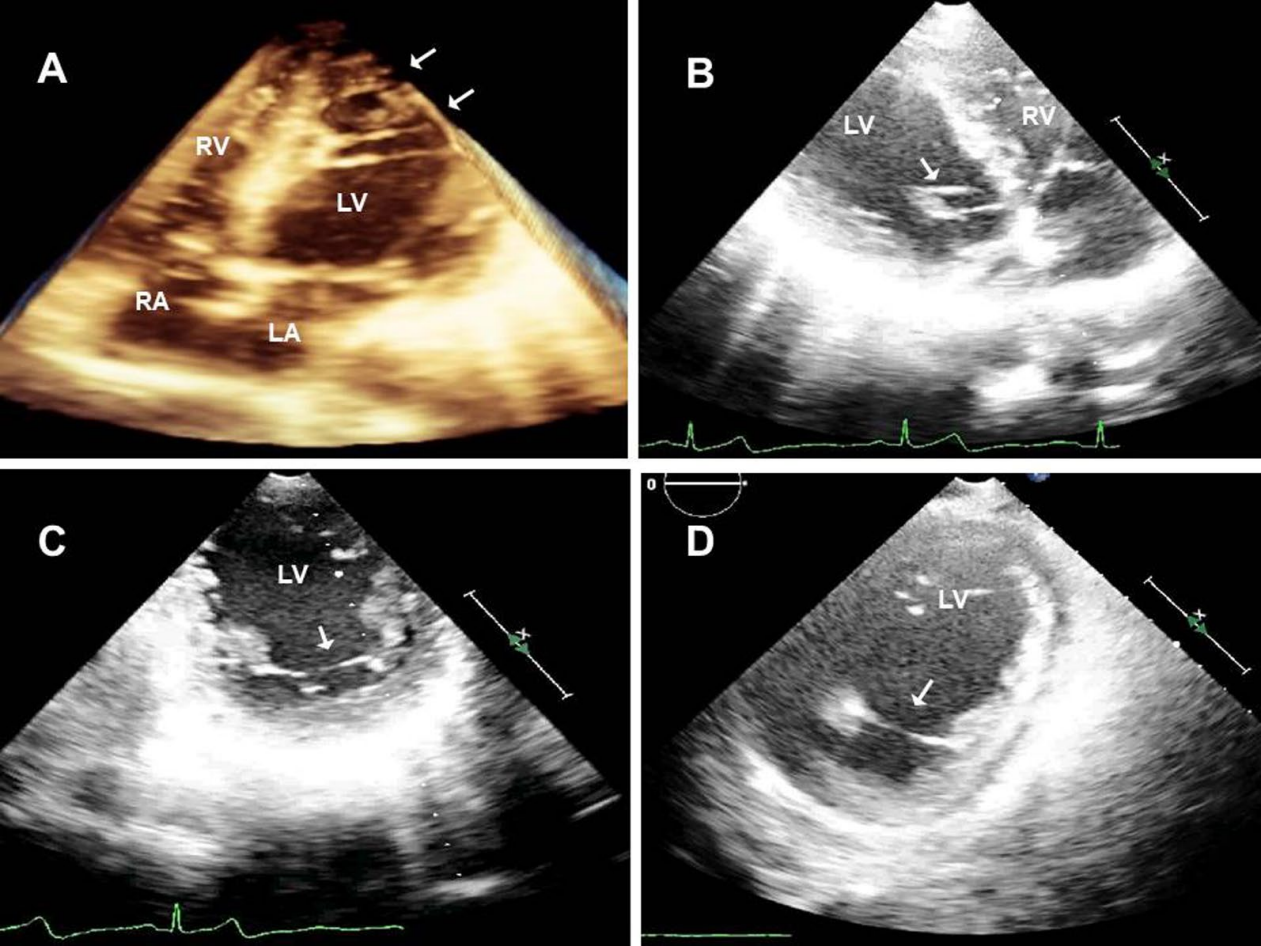
**A** Apical four-chamber three-dimensional transthoracic echocardiographic view with the appearance of multiple left ventricular false tendons (arrows). **B** Modified Four-chamber long-axis view depicting two left ventricular false tendons (arrow) from anterolateral mitral papillary muscle to basal segment of the anterior septal wall. Left ventricular false tendons are restricting the movement of the papillary muscle by pulling toward to interventricular septal wall. Fortunately, mitral valve dysfunction did not emerge secondary to these lesions. **C**, **D** Modified apical two-chamber view revealed complex branching left ventricular false tendons (arrow) between the base of anterolateral and posteromedial mitral papillary muscles and chordae tendinea

Left ventricular false tendon (LVFT) is a thin, ribbon-like, fibrous, and/or muscular structure that extends from the septum to the left ventricle posterior or lateral wall or both [6] (Fig. 1, Additional file 1: Video 1). It can be observed between papillary muscles or between papillary muscles and the left ventricle wall [7]. And they can be found single or multiple (Fig. 2, Additional file 2: Video 2). In embryological life, heart muscle develops from the inner and outer layers. LVFT and trabecular structures grow out of the inner layer. Unlike trabeculae carneae, papillary muscle, and chordae tendineae, LVFT extends as a band between left ventricle walls [8]. It is rarely detectable on routine two-dimensional transthoracic echocardiography (TTE) examination. Particularly modified images are usually required [9]. And it is best detected on apical long-axis images. In general, it has no clinical significance [10]. Robert et al. showed that turbulence created by LVFT in the ventricle causes innocent vibration murmur [11]. Another study observed that the severity of mitral regurgitation decreased in patients with LVFT in dilated cardiomyopathy [12]. However, in a study positive correlation was found between LVFT and ventricular premature beats conducted in healthy individuals [13]. And, some studies revealed that LVFT is effective in the occurrence of ventricular tachycardia [14, 15]. Also, LVFT ablation has been beneficial in the termination of narrow QRS tachycardia [16]. Furthermore, Nakagawa et al. observed that the J wave, which is known to be associated with the formation of ventricular fibrillation, was detected more frequently in patients with LVFT [17]. These studies are based on the stimulation of purkinje fibers in the structure of LVFT during the mechanical tension of the left ventricle wall. In addition to the arrhythmic effects of LVFT, rupture of LVFT due to surgery, ischemic or dilatation may also cause contraction defects [18]. Moreover, some case studies of infective endocarditis localized in LVFT contribute to the clinical importance of LVFT [19].

Three-dimensional echocardiographic evaluation to overcome many of the disadvantages of two-dimensional echocardiography has been fully recognized. Cardiac structural disorders can be evaluated better with three-dimensional TTE than with traditional two-dimensional TTE [20–22]. Three-dimensional echocardiography allows improvement in the accuracy and reproducibility of the evaluation of cardiac abnormalities by eliminating the need for geometric modeling and the errors caused by foreshortened views. Three-dimensional echocardiography provides more realistic visualization of cardiac lesions [23]. Therefore, diagnosis of LVFT cannot be made with certainty by routine two-dimensional TTE [9]. In our study, we examined the coexistence of two congenital anatomical variants using the three-dimensional TTE method.

There are few studies about CN or LVFT in the literature. And, to our best knowledge, there is no study conducted on the coexistence of these two congenital residues.

## Methods

### Study design and population

We conducted a single-center, prospective study with CN cases who were admitted to the cardiology polyclinic with various complaints. Patients’ clinical, laboratory, electrocardiography, and echocardiography findings were recorded.

In the echocardiography evaluation of 12,171 patients, CN was detected in 49 cases. All possible cases of CN were included based on a cardiologist-approved algorithm (at least post-fellowship experience of 5 years). If insufficient information was available to apply the definition criteria, the event was excluded from the study. All confirmators, reviewers, and abstracters were blinded concerning the study. A detailed physical examination of all patients was performed. Twelve-channel electrocardiography and standard TTE images were obtained. Six of the patients were excluded from the study because of the lacking blood tests.

### Echocardiographic analysis

Echocardiography examination (transthoracic and transesophageal) was performed with a Philips Medical Systems iE33xMATRIX (Bothell, WA, USA) echocardiography device with a 1–5 MHz X5-1 probe. TTE images were examined following the guidelines [24].

In our study, all patients with CN were also evaluated with real-time three-dimensional TTE. Following the literature [25], the conditions of bilateral echo-free space and loosening of the tendon in the systolic phase were sought in the echocardiographic images to distinguish LVFT from other structures of the heart.

A contrast echocardiographic examination was performed in patients with IASA or suspicious Doppler flow in the interatrial or ventricular septum. The contrast echocardiographic procedure was performed by applying agitated saline infusion. The procedure was repeated by performing the valsalva maneuver during the procedure in patients whose transition was not observed in routine practice. Transesophageal echocardiography (TEE) was performed in patients with positive contrast echocardiography for assessment of the anatomy of the atrial and ventricular septum.

### Ethics statement

The study data were evaluated by double-blinded consultant cardiologists (at least post-fellowship experience of 5 years).

### Statistical analysis

While evaluating the findings obtained in the study, “SPSS for Windows 16.0” statistical package program (SPSS Inc, Chicago, IL, USA) was used for statistical analysis. Since continuous variables were normally distributed as mean ± standard deviation and categorical data were expressed as percentages. The compliance of the data to the normal distribution was evaluated with the Kolmogorov–Smirnov test. The assumption of normal homogeneity of the data was determined by the ANOVA (one-way analysis of variance) test. Student *t* test was used in the analysis of non-categorical data. Correlations of nonparametric data were investigated by Spearsman and the correlation of parametric data with Pearson correlation analysis. Independent sample *t* test was used to compare the mean values of two separate groups. Variance analysis was used to identify different groups. The Chi-square test was used to evaluate categorical variables. Comparison of the frequency of LVFT found as a result of studies in the literature and the frequency of LVFT in patients with CN in our study was performed using the Chi-square test. For statistical significance *p*-value < 0.05 was accepted.

## Results

In our study, CN was found in 49 (0.4%) of 12,171 patients who were evaluated with two-dimensional TTE in the routine cardiology polyclinic examination. The distribution of the patients by gender was 16 (32.7%) females (age: 35.1 ± 15.3 years) and 33 (67.3%) males (age: 36.2 ± 15.1 years). Demographic, echocardiographic, and laboratory data of the study group are listed in Table 1. Normal sinus rhythm was observed on electrocardiography examinations. No early beats, bundle branch block, or any rhythm disturbances were observed. On TTE examination, only one patient had a left ventricle ejection fraction (LVEF) below the normal limits (LVEF: 47%).

**Table 1:** Demographic, echocardiographic, and laboratory findings of the patients with Chiari network

Characteristic	*n* = 49	%	Mean ± standard deviation
Gender (male)	33	67.3	
Age (years)			35.9 ± 15.0
Body mass index (kg/m^2^)			22.8 ± 3.1
Systolic blood pressure (mmHg)			117.6 ± 9.7
Heart rate (beats/minute)			68.9 ± 9.7
Hypertension	5	10.2	
Dyslipidemia	4	8.2	
Diabetes mellitus	5	10.2	
Chronic kidney disease	1	2.0	
Smoking	21	42.9	
Sodium (mmol/L)			140.0 ± 2.2
Potassium (mmol/L)			4.4 ± 0.4
Creatinine (mg/dL)			0.9 ± 0.2
Glucose (mg/dL)			97.1 ± 44.6
AST (U/L)			19.5 ± 6.3
ALT (U/L)			20.0 ± 13.9
*Echocardiography parameters*			
Aortic root diameter/Left atrium (cm)			2.7 ± 0.4/3.2 ± 0.4
Right atrium/RVDd (cm)			3.0 ± 0.4/2.8 ± 0.4
IVSd/LVPWd (cm)			0.9 ± 0.2/0.9 ± 0.2
LVIDd/LVIDs (cm)			4.5 ± 0.5/2.9 ± 0.4
E/A (cm/s)			0.8 ± 0.2/0.6 ± 0.1
LVEF (%)			64.9 ± 5.8
Aortic insufficiency (mild)	2	4.1	
Mitral insufficiency (mild)	16	32.7	
Tricuspid insufficiency (mild or moderate)	19	38.7	
Pulmonary insufficiency (mild)	2	4.1	
IASA	31	63.3	
Positive contrast echo	22	44.9	
Atrial septal defect	2	4.1	
Patent foramen ovale	4	8.2	

Chronic kidney disease—creatinine > 2.0 mg/dl; hemodialysis or renal transplantation

*ALT* Alanine transaminase; *AST* Aspartate transaminase; *IASA* Interatrial septal aneurysm; *IVSd* Interventricular septal end-diastole diameter; *LVEF* Left ventricle ejection fraction, *LVIDd* Left ventricular internal end-diastole diameter; *LVIDs* Left ventricular internal end-systole diameter; *LVPWd* Left ventricular posterior wall end-diastole diameter; *RVDd* Right ventricular end-diastole diameter

LVFT was observed in 31 (63.3%) of 49 CN patients. In the literature, the frequency of LVFT rate was found to be very variable (0.2%–78%) (Table 2). Among the existing studies, the ratio of LVFT was calculated as 22% in 15 studies that examined the frequency of LVFT by the two-dimensional TTE (Table 3). Hence, the frequency of LVFT in our study population was significantly higher than the average ratio of the literature studies (%63.3 versus 22%) (p = 0.01) (Table 4).

**Table 2:** The ratios of the left ventricular false tendon in the literature

Year	Authors	Age group	Study population	Detection method	LVFT ratio n (%)
1981	Okamoto et al. [39]	All ages	132	2D-Echo	61 (46)
1981	Nishimura et al. [40]	All ages	1000	2D-Echo	5 (0.5)
1983	Perry et al. [41]	≤ 15	3847	2D-Echo	31 (0.8)
1984	Sethuraman et al. [42]	> 12	1012	2D-Echo	4 (0.4)
1984	Ryssing et al. [31]	All ages	2000	2D-Echo	4 (0.2)
1984	Vered et al. [43]	All ages	2079	2D-Echo	42 (2)
1984	Suwa et al. [36]	> 12	1117	2D-Echo	71 (6)
1984	Brenner et al. [44]	≤ 18	100	2D-Echo	61 (61)
1984	Keren et al. [25]	≥ 15	35	2D-Echo	15 (43)
1984	Gerlis et al. [33]	> 18	800	2D-Echo	4 (0.4)
1986	Luetmer et al. [7]	All ages	483	Autopsy	265 (55)
1986	Casta et al. [45]	Children	218	2D-Echo	31 (14)
1986	Malouf et al. [38]	All ages	488	2D-Echo	123 (25)
1987	Boyd et al. [46]	All ages	474	Autopsy	322 (68)
1990	Abdulla et al. [35]	All ages	100	Autopsy	34 (34)
1992	Cocchieri et al. [34]	< 13	273	2D-Echo	80 (29)
2003	Kervancioglu et al. [9]	< 10	368	2D-Echo	97 (26)
2011	Philip et al. [32]	< 18	476	2D-Echo	371 (78)

2*D-Echo* Two-dimensional transthoracic echocardiography; *LVFT* Left ventricular false tendon

**Table 3:** The ratios of the left ventricular false tendon in the literature are detected only by two-dimensional transthoracic echocardiography

Year	Authors	LVFT ratio (%)
1981	Okamoto et al. [39]	46
1981	Nishimura et al. [40]	0.5
1983	Perry et al. [41]	0.8
1984	Sethuraman et al. [42]	0.4
1984	Ryssing et al. [31]	0.2
1984	Vered et al. [43]	2
1984	Suwa et al. [36]	6
1984	Brenner et al. [44]	61
1984	Keren et al. [25]	43
1984	Gerlis et al. [33]	0.4
1986	Casta et al. [45]	14
1986	Malouf et al. [38]	25
1992	Cocchieri et al. [34]	29
2003	Kervancioglu et al. [9]	26
2011	Philip et al. [32]	78

The compliance of the literature population to normal distribution was evaluated with the Kolmogorov–Smirnov test. Since the general population data were normally distributed, mean values were expressed

*LVFT* Left ventricular false tendon

**Table 4:** Comparison of the left ventricular false tendon ratio in literature and patients with Chiari's network

	Patients with Chiari’s network (%)	The average ratio of literature studies (%)	*P*-value
Left ventricular false tendon	63.3	22	0.01

A Chi-square test was used for comparison

*P* < 0.05 was considered significant

Thirty-one (63.3%) patients had an IASA. And, contrast echocardiography examination was positive in 22 (61.1%). As a result of the TEE examination of ten patients, ASD was observed in two patients and a PFO was observed in four patients. Also, cor triatriatum sinister was detected in one patient and membranous ventricular septal defect (VSD) in one patient. No relation was observed between the presence of LVFT and the frequency of IASA and positive echo-contrast examination (respectively *p* = 0.40, 0.09). Also, no association was found between echocardiographic parameters (aortic root diameter, left atrium diameter, right atrium diameter, right ventricular internal end-diastole diameter, interventricular septal end-diastole diameter, left ventricular posterior wall end-diastole diameter, left ventricular internal end-diastole diameter, left ventricular internal end-systole diameter, LVEF) and LVFT (respectively *p* = 0.50, 0.38, 0.13, 0.34, 0.70, 0.68, 0.28, 0.49, 0.90).

## Discussion

In terms of gender, male dominance (67.3%) was present. And the study population was generally young. Also, the mean body mass index was within the ideal limits. Our patient group had a percentage close to normal population rates in terms of chronic disease history (coronary artery disease, hypertension, dyslipidemia, diabetes mellitus, and thyroid dysfunction). Hence, the majority of patients in our study consisted of healthy individuals with no history of functional cardiac abnormalities. Exceptionally, smoking was found at a high rate (44.5%).

In autopsy studies, the ratio of CN was found to be 1.3–4% [26–28]. In TTE studies, the ratio ranges between 0.03 and 9.5% [29, 30]. Case limitation is the biggest problem in studies. Also, the reason for the ratio differences in the studies may be caused by the difference in the population included in the studies or the visual similarity between CN and the Eustachian valve [29]. In our study, the incidence of CN was found to be 0.4%, consistent with the literature.

In studies, the frequency of LVFT was found within widely ratios (0.2%–78%) (Table 2) [31, 32]. Imaging quality, study plan (TTE, autopsy), selection of study population (child, adult), the difference in number and quality of TTE image windows, and the difference in the criteria used for LVFT definition may have caused this difference [7]. In autopsy studies, where the current disadvantages were minimal, significantly higher LVFT rates could be detected rather than in TTE studies [33, 34]. In a study that aims to compare TTE and autopsy determination, LVFT was found in 18% of TTE examinations and 34% of autopsy examinations in the same population [35]. In Keren et al.'s study, echocardiographic evaluation before and after cardiac transplantation and post-autopsy was compared in terms of detecting LVFT, and the sensitivity of the echocardiographic evaluation was 85% and the specificity was 82%. An increase in the frequency of LVFT was observed in new echocardiography studies. The underlying causes may be the emergence of new echocardiography methods and the improvement of the image quality of echocardiography devices. Hence, we evaluated CN patients in detail by three-dimensional TTE (Fig. 1, Additional file 1: Video 1).

**Fig. 3:**
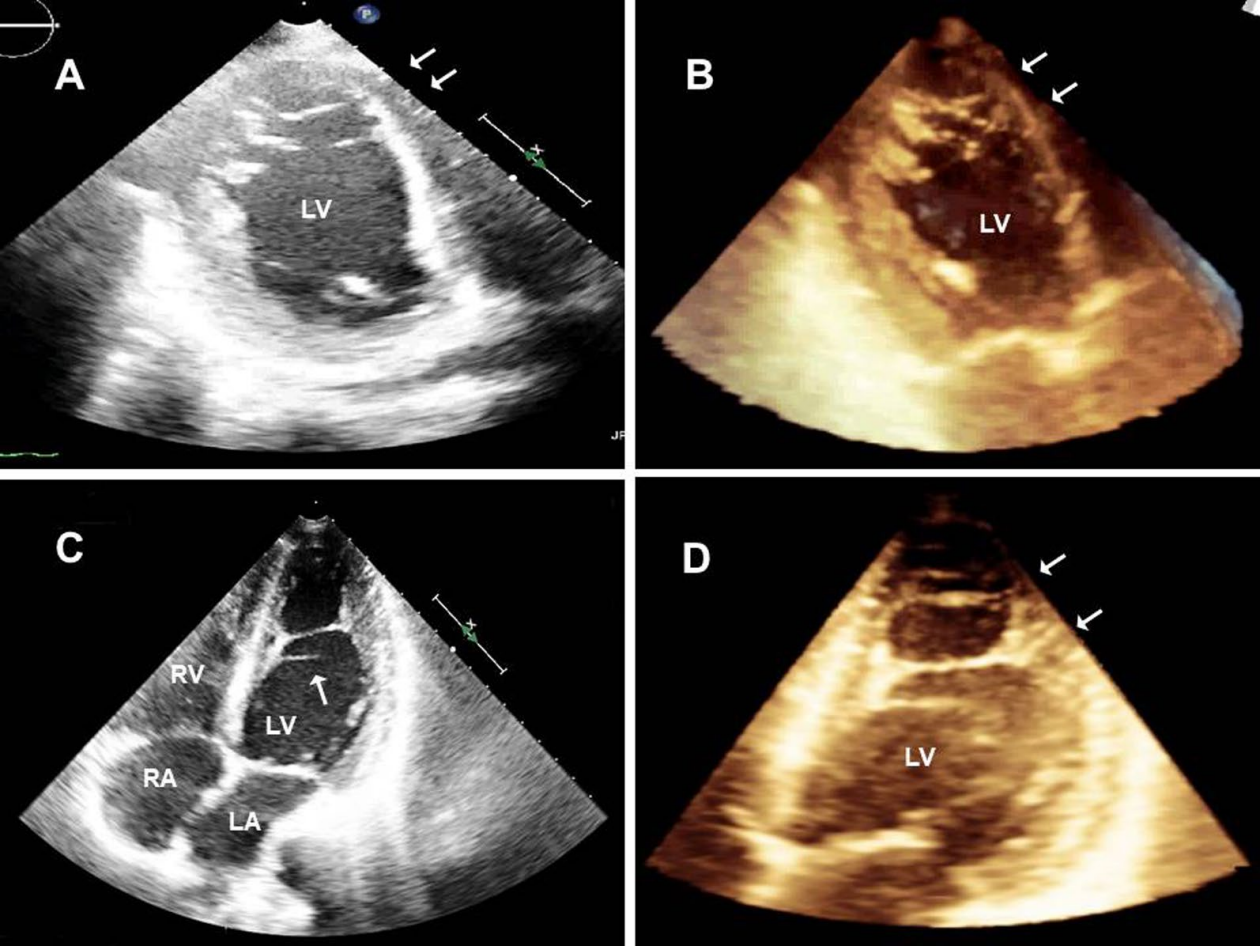
**A**, **C**. Modified two-dimensional echocardiographic examination of two patients shows only two left ventricular false tendons (arrows). However, **B**, **D** three-dimensional examination of the same images reveals multiple, mesh-shaped, complex left ventricular false tendon structures (arrows)

In our study, the problem of including the LVFT image window was encountered, and it was tried to be determined with the help of modified and three-dimensional TTE images. In our population, multiple, complex LVFT structures could not be detected by a standard two-dimensional TTE view. Further examination with three-dimensional TTE revealed multiple, mesh-shaped, complex LVFT structures (Fig. 3). Among the existing 15 two-dimensional TTE studies, the ratio of LVFT was calculated only as 22% (Table 3). These results may have been less than the actual rates, because of limited examination of two-dimensional TTE. Compatibly, these rates were far less than our three-dimensional TTE evaluated LVFT ratios (63.3%). Three-dimensional TTE was more useful in detecting the LVFT than two-dimensional TTE.

Although the frequency of LVFT in our series detected was high, it may have been detected at a much lower rate than actually, it should have been. Conversely, our study population was young and had normal BMI and cardiac function. Thus, the appearance of the LVFT was facilitated by the good quality of the TTE images. In this respect, it would be more appropriate to evaluate similar studies in the autopsy series.

No relation was found between the frequency of LVFT and gender (*p* = 0.48). In the literature, the incidence of LVFT was higher in males [6, 7, 36]. The reason for no gender difference was observed in the frequency of LVFT in our study population with CN; it may be due to the small number of females enrolled in the study or the fact that insufficient image quality of females. Otherwise, our results are consistent with the Framingham Study (*p* = 0.99 for gender difference) [10].

In our study, no premature contractions were observed in the electrocardiography records of any patient with LVFT. However, ventricular premature beats have been found to be associated with LVFT in several studies [13, 36, 37]. The fact that we did not perform rhythm Holter monitoring prevents us from commenting on the frequency of arrhythmia in our study.

The ratio of LVFT in our population with CN (63.3%) was found to be significantly higher than the studies in the literature (22%) (Table 4) (*p* = 0.01). As a result, it was revealed that two cardiac variables such as CN and LVFT may be related in our study. Similarly, the association of LVFT with other cardiac pathologies has been shown in previous studies. No cardiac problems were detected in 23% of cases with LVFT [38].

Although it has been shown in the literature that LVFT is associated with infective endocarditis, mitral valve prolapse, mitral regurgitation, and even CN is associated with ASD, IASA, and PFO, these cardiac variant structures are ignored in the echocardiography examination for clinicians. Our study reveals the coexistence of two cardiac variants. Thus, it supports the possibility of the two structures accompanying other cardiac variant structures and suggests that they should be evaluated more carefully in the echocardiography examination as they can be in synergy in terms of the cardiac pathologies they accompany.

### Study limitations

Since it is difficult to accumulate the number of cases in a single center for CN, which is a rare abnormality. Newly planned, multi-center studies with a high number of cases will be valuable.

Also, lower age and the predominance of the male gender are the limitations of the study.

Furthermore, ASD, PFO, and VSD ratios in our study may have been less than the actual rates. Because, we were able to perform TEE only on patients with positive contrast echocardiography, not on all the CN patients. And, we were able to perform TEE on only half of the patients with positive contrast echocardiography, because of the failure to obtain a consent form. Therefore, VSD, PFO, or ASD could not be evaluated in all CN patients.

## Conclusions

The presence of CN increases the probability of coexistence of LVFT in relevant patients compared to the normal population. And also, cardiac anomalies like an ASD and IASA are frequently associated with CN. CN patients need to be examined in detail. LVFT can be evaluated better with three-dimensional TTE than with traditional two-dimensional TTE. And patients with CN should be evaluated more carefully by three-dimensional echocardiography as they can be in synergy in terms of the cardiac pathologies they accompany. With the frequent use of echocardiography, the development of new echocardiography devices and imaging techniques, LVFT anatomy, CN structure, and their relationship with other cardiac structures will be better demonstrated.

## Supplementary Information

**Additional file 1 ** The apical four-chamber imaging shows the Chiari’s network movement like a whip inside the right atrium and the left ventricular false tendon like a band form inside the left ventricle.

**Additional file 2** *Case 1* Three-dimensional apical four-chamber view showed left ventricular false tendons between the mid-apical septum to the anterolateral wall.* Case 2A* Three-dimensional modified apical two-chamber view revealed left ventricular false tendons between anterolateral and posteromedial mitral papillary muscles and chordae tendinea. *Case 2B* Three-dimensional modified parasternal long-axis view depicting two left ventricular false tendons from the anterolateral papillary muscle to the septal wall. Left ventricular false tendons are restricting the movement of the papillary muscle by pulling toward to septal wall. Fortunately, mitral valve dysfunction did not observe secondary to these lesions. *Case 3* Three-dimensional video of complex branching of multiple left ventricular false tendons (network-like).

## Data Availability

All data analyzed during this study are included in this article. And all data are available on request at the archive of the Department of Cardiology University of Health Sciences Turkey, Sisli Hamidiye Etfal Training and Research Hospital, Information Management Database.

## Abbreviations

ASD: Atrial septal defect
CN: Chiari’s network
LVFT: Left ventricular false tendon
IASA: Interatrial septal aneurysm
LVEF: Left ventricle ejection fraction
PFO: Patent foramen ovale
TEE: Transesophageal echocardiography
TTE: Transthoracic echocardiography
VSD: Ventricular septal defect
